# The T4/T3 quotient as a risk factor for differentiated thyroid cancer: a case control study

**DOI:** 10.1186/s40463-017-0208-0

**Published:** 2017-04-04

**Authors:** Mark Sasson, Emily Kay-Rivest, Rami Shoukrun, Anca Florea, Michael Hier, Veronique-Isabelle Forest, Michael Tamilia, Richard J. Payne

**Affiliations:** 1 McGill University, 3655 Sir William Osler, Montreal, H3G 1Y6 QC Canada; 2 Department of Pathology, Jewish General Hospital, 3755 Côte-Sainte-Catherine, Montreal, H3T 1E2 QC Canada; 3 Department of Otolaryngology Head and Neck Surgery, Jewish General Hospital, 3755 Côte-Sainte-Catherine, Montreal, H3T 1E2 QC Canada; 4 Division of Endocrinology and Metabolism, Jewish General Hospital, Jewish General Hospital, 3755 Côte-Sainte-Catherine, Montreal, H3T 1E2 QC Canada

**Keywords:** Serological markers, Thyroid malignancy, Thyroxine, Triiodothyronine

## Abstract

**Background:**

The incidence of thyroid nodules is increasing among patients in North America. Few of these nodules harbour malignancy, thus further research is required to identify predictive markers of malignant thyroid disease. This study set out to understand the relationship between the levels of fT4 and fT3 and differentiated thyroid cancer.

**Methods:**

A case-control study was conducted with 142 cases and 86 controls from the McGill University Teaching Hospitals. All patients underwent thyroid surgery. Cases were defined as patients with malignant nodules confirmed on final pathology and controls were defined as patients with benign nodules. The serological levels of TSH, fT4 and fT3 were measured preoperatively. Odds ratios were determined for each parameter and logistic regressions were calculated between markers and probability of malignancy. Additionally, fT4 values were divided by fT3 values (fT4/fT3 quotient) for each patient and an odds ratio was calculated.

**Results:**

Amongst cases, the mean TSH was 2.25 ± 0.360U/mL, fT4 was 14.8 ± 0.689pmol/L, and fT3 was 4.65 ± 0.463pmol/L. Amongst controls, the mean TSH was 2.36 ± 1.68U/mL, fT4 was 14.3 ± 1.71pmol/L, and fT3 was 5.27 ± 0.957pmol/L. Patients in the control group were more likely to have low TSH, while patients in the case group would have high fT4 and patients in the control group were more likely to have a low fT4. The OR for patients with TSH >4.4U/mL was 2.13 (0.97, 4.65), and for patients with TSH <0.4U/mL was 0.46 (0.22, 0.95). The OR for patients with fT4 > 16pmol/L was 2.10 (1.09, 4.06), and for patients with fT4 < 10pmol/L was 0.45 (0.20, 0.98). The OR for patients with fT3 > 5.5pmol/L was 0.39 (0.14, 1.28). The OR for patients with fT3 < 3pmol/L was 1.83 (0.25, 13.69). The average fT4/fT3 was 3.39 ± 0.206 for cases and 2.93 ± 0.467 for controls. The fT4/fT3 quotient was considered high if it was >3.3 (OR =6.00 (2.94, 12.25)).

**Conclusion:**

In this study, a direct relationship between high levels of fT4 and malignancy was uncovered. Furthermore, low levels of TSH and fT4 increased the likelihood that a nodule was benign. In this study a fT4/fT3 ratio >3.3 increased the risk of malignancy by 3.6 times (*p*-value =0.0013).

## Background

The incidence of thyroid nodules has been increasing [1]. It is believed that approximately 5–15% of these nodules are malignant [2–4]. While thyroid malignancies make up only the minority of these nodules, they are becoming more common in Canada and the United States [5–7]. This increase is likely in part due to increased detection rates through the use of ultrasound.

It has been well documented that there is a relationship between levels of thyroid stimulating hormone (TSH) and thyroid malignancy. A TSH level that is above the normal range or even in the upper range of normal is thought to increase the likelihood that a thyroid nodule is malignant [8–10]. It stands to reason that if the levels of TSH are elevated in patients with thyroid cancer, it is possible that elevated levels of thyroxine (T4) and triiodothyronine (T3) may also be correlated with malignancy as well. Cho et al. has shown that free T4 (fT4) is elevated in patients with thyroid cancer (odd’s ratio (OR) of 1.73) [11]. However, the inverse has been shown to be true for total T3 (TT3) by Jonklaas et al. [12]. To the best of our knowledge, there is no evidence-based hypothesis which explains this relationship.

Thyroid nodules are commonly imaged by ultrasound. This technique can help determine the size of a nodule, which along with increased age and male sex, is associated with thyroid cancer. Ultrasound features are important in determining the need for a thyroid biopsy [13]. Indeed, the gold standard for diagnosing thyroid malignancies is ultrasound guided fine-needle aspiration biopsy (USFNA), [14] with a diagnostic sensitivity of 65–98% and specificity of 72–100% [15]. Nevertheless, USFNA is not perfect, [16, 17] and appropriate management of these nodules may not be obvious [18, 19]. In cases where the USFNA yields indeterminate results, serological markers could play a role as a predictor of thyroid nodule malignancy.

By studying serological data taken from patients who have undergone thyroid surgery, we hope to better understand the relationship between the levels of fT4 and fT3 and differentiated thyroid cancer which can be used as a clinical tool to help understand the true nature of indeterminate thyroid nodules. Furthermore, the goal of this study was to provide further evidence in understanding whether fT4 and fT3 increase or decrease in the likelihood of malignancy, helping to clarify the discrepancy between Cho et al. and Jonklaas et al.

## Methods

A retrospective chart review of patients who underwent thyroid surgery was performed. Patients undergoing thyroid surgery at the McGill University Teaching Hospitals between January 2014 and October 2015 were included. Ethics approval was obtained by the Ethics Review Board of the Jewish General Hospital and the Research Institute of the McGill University Health Centre. Patients who previously underwent hemithyroidectomies were excluded. Patients with a diagnosis of papillary microcarcinoma on final pathology were excluded. Patients whose blood tests were measured >3 months prior to surgery were excluded.

Malignancies that were considered in the cases group were papillary thyroid cancer and follicular carcinoma. Cases that were considered benign included follicular adenoma, colloid cysts, Hurthle cell adenoma, Hashimoto’s thyroiditis, and nodular hyperplasia.

Patients were not required to have had all three values (TSH, fT4 and fT3) measured to participate in this study. If they did not have a given value, the patient was treated as if they did not exist for all calculations relating to said value. To clarify, if a patient had TSH and fT4 values, they were included in all calculations relating to TSH and fT4, but treated as if they did not exist for any calculations relating to fT3.

The reference ranges at our hospital are 0.4–4.5 I/mL for TSH, 9.0–26.0 pmol/L for fT4 and 2.8–7.1 pmol/L for fT3. If multiple values were recorded in this time frame, the average value was used for the purposes of all calculations. Given the wide range of these reference ranges, it was decided to narrow these ranges in the data analysis. Indeed, in the methodology of Cho et.al. fT4 was only considered high if patients were in the highest tertile of recorded values and not based on the normal reference lab ranges [11]. For this reason, it was decided that the normal reference ranges would be such that 80% of cases and controls were within the range for TSH and fT4, and 90% of cases and controls were within the range for fT3. It was decided to include fT3 values as a case only if further away from the mean, since fT3 is known to be a more variable test [20]. TSH, fT4, and fT3 values were measured with an immunometric assay performed on the Elecsys 2010 Immunoassay Analyzer (Roche Diagnostics). All statistical analysis was performed in Microsoft Excel using the basic program and the Solver add-on.

In the initial data analysis, cases were directly compared to controls without stratification. Following this, ORs were calculated for gender, past history of cancer, past history of thyroid disease and both low and high serum levels of TSH, fT4 and fT3. Furthermore, patients with nodules measuring 2–2.9 cm, 3–3.9 cm, and >3.9 cm were then isolated in the data and compared on all the aforementioned qualities to the controls. These cut-offs were set according to the cut-offs in the McGill Thyroid Nodule Score (MTNS) [21] in order to compare large nodules to the average sized nodules. All ORs were calculated with a 1-tailed 95% confidence intervals.

Values were considered significant if the confidence interval did not cross 1 and the *p*-value was <0.05. Additionally, two univariate logistic regressions were performed between malignancy against fT4 and fT3 respectively to determine if there was a linear relationship between the probability of a nodule being malignant and the levels of these thyroid hormones.

For each case and control for whom both fT4 and fT3 values were available, a quotient was calculated. If the quotient was over 3.3, it was considered high. This cut off was set by taking a high fT4 value of 18.5 and dividing it by a high fT3 value of 5.5.

## Results

633 patients underwent surgery between January 2014 and October 2015 by a single surgeon. Of these, 228 patients met the inclusion criteria. This resulted in a cohort of 228 patients, 142 cases of patients with malignancies and 86 controls of patients with benign nodules. The average age of all patients in the study was 51. The study consisted of 58 men and 170 women. See Tables 1 and 2 for further demographic information. Values were considered normal if they were within the following ranges: 0.4–4.5 U/mL for TSH, 10.0–16.0 pmol/L for fT4 and 3.0–5.5 pmol/L for fT3.

**Table 1:** Table of descriptors

	All	Cases	Controls
Patients (*n*)	228	142	86
Age	51	51	52
Male (*n*)	58	39	19
Male (%)	25	27	22
Female (*n*)	170	103	67
Female (%)	75	73	78
Past History of Thyroid Disease (*n*)	27	15	12
Past History of Thyroid Disease (%)	12	11	14
Past History of Malignancy Disease (*n*)	9	6	3
Past History of Malignancy Disease (%)	3	4	3

**Table 2:** Table of thyroid descriptors

	Cases			Controls			*p*-value
	Average	Confidence interval		Average	Confidence interval	Amount	
All Patients							
TSH	2.25	0.360	140	2.36	1.68	84	0.874
fT4	14.8	0.689	131	14.3	1.71	77	0.478
fT3	4.63	0.465	80	5.27	0.946	48	0.199
fT4/fT3	3.85	0.206	75	2.94	0.467	42	0.047
2−2.9 cm Nodule							
TSH	2.42	0.77	40	1.98	0.55	20	0.089
fT4	15.24	1.53	37	13.08	1.28	18	0.079
fT3	5.30	1.73	20	4.86	0.23	11	0.713
fT4/fT3	3.47	0.47	18	2.85	0.33	9	0.096
3−3.9 cm Nodule							
TSH	2.37	0.74	30	1.70	1.02	20	0.283
fT4	14.83	1.71	27	13.84	0.97	18	0.388
fT3	4.39	0.38	14	4.74	0.41	11	0.223
fT4/fT3	3.36	0.59	13	3.10	0.49	11	0.520
>3.9 cm Nodule							
TSH	2.64	1.18	25	1.64	0.41	20	0.202
fT4	15.10	1.47	24	14.36	0.64	17	0.475
fT3	4.62	0.63	13	5.15	0.26	10	0.233
fT4/fT3	3.35	0.40	12	2.97	0.17	9	0.133

For the cases, the number of patients who had TSH, fT4 and fT3 values that met the inclusion criteria were 140, 131 and 80 respectively. For the controls, the number of patients who had TSH, fT4 and fT3 values that met the inclusion criteria were 84, 77 and 48 respectively. The range of values were 0–68.97 U/mL for TSH, 2.2–61.6 pmol/L for fT4 and 2.3–26.9 pmol/L for fT3. See Table 2. The mean values and confidence intervals for the stratifications based on size are also included in Table 2. The mean values of the fT4/fT3 quotient can be found here as well.

Patients in the case group were more likely to have high fT4, while patients in the control group were more likely to have a low TSH and fT4. See Table 3 for more information. The ORs for the stratifications based on size are also included in Table 3, though none yielded significant results. ORs that could not be calculated due to a lack of patients with abnormal values were not included in Table 3. The OR of the fT4/fT3 quotient can be found here as well. The sensitivity of the fT4/fT3 quotient calculation using the cut off of >3.3 as high as 0.48 and the specificity is 0.867.

**Table 3:** Table of TSH, fT4 and fT3 ORs. ORs that could not be calculated due to a lack of patients with abnormal values were not listed below

All Nodules	OR	CI −	CI+	*p*-value
TSH > 4	2.13	0.97	4.66	0.0666
TSH < 0.4	0.46	0.22	0.95	0.0500
fT4 > 16	2.10	1.09	4.06	0.0339
fT4 < 10	0.45	0.20	0.98	0.0603
fT3 > 5.5	0.39	0.14	1.12	0.107
fT3 < 3	1.83	0.25	13.69	0.605
fT4/fT3 > 3.3	6.00	2.56	14.07	0.0001
2–2.9 cm Nodules	OR	CI+	CI −	*p*-value
fT4 > 16	2.57	0.60	11.04	0.262
fT4 < 10	0.44	0.10	2.00	0.349
fT4/fT3 > 3.3	5.20	1.13	23.89	0.0610
3 –3.9 cm Nodules	OR	CI+	CI −	*p*-value
TSH > 4.4	4.75	0.68	33.14	0.165
TSH < 0.4	1.13	0.29	4.48	0.875
fT4 > 16	2.96	0.40	22.105	0.351
fT4 < 10	2.13	0.27	16.87	0.529
fT4/fT3 > 3.3	5.83	1.12	30.37	0.0640
>3.9 cm Nodules	OR	CI+	CI −	*p*-value
TSH < 0.4	0.78	0.13	4.85	0.234
fT4 > 16	0.93	0.22	4.02	0.935
fT3 > 5.5	0.70	0.06	8.69	0.803
fT3 < 3	0.75	0.06	9.87	0.846
fT4/fT3 > 3.3	9.60	1.16	79.41	0.0640

The univariate logistic regression revealed a linear relationship between fT4 levels and the probability of developing cancer. The slope and intercept of the line were 0.0224 and 0.2658 respectively. The range is from 57–84%. The R^2^ of the line was 0.9229. The univariate logistic regression also revealed a linear relationship between fT3 levels and the probability of developing cancer. The slope and intercept of the line were −0.0868 and 0.8721 respectively. The range is from 19–66%. The R^2^ of the line was 0.999.

## Discussion

This study compared the TSH, fT4 and fT3 values of patients with thyroid malignancies to those with benign thyroid nodules. Given that there is still controversy in the literature regarding how these thyroid hormones change in the context of malignancy, [11, 12] this study attempts to provide further insight into better understanding the true relationship between these hormones and thyroid cancer. Additionally it attempts to set a cut-off quotient of fT4/fT3 > 3.3 at which the likelihood of malignancy increases. Though further research is required to demonstrate the extent of this relationship, in this study, the quotient has been found to be strongly correlated with malignancy. Finally, this study observes size of nodules in cases of malignancy and attempts to determine whether TSH, fT4 or fT3 change with thyroid nodule size.

To date, several studies have described that elevated TSH levels may be associated with thyroid malignancy. Though the average TSH values for patients with thyroid malignancy in this study was similar to the control group, the odd’s ratio of high TSH corresponding to malignancy is in accordance with previous studies [9, 10, 21–24]. Despite the association between elevated TSH and malignancy, the TSH levels in both groups are similar (*p*-value of 0.874).

The OR calculated in patients with thyroid nodules of all sizes supports the initial hypothesis that patients without thyroid malignancy are less likely to have higher levels of fT4. This is complementary to the previously conducted study by Cho et al. [11]. However, this is not in agreement with Jonklaas et al. who have found no significant relationship between these variables [12]. This relationship can be explained simply as Jonklaas only considered patients to have high fT4 when it was greater than 23.2 pmol/L (1.80 ng/dL.) Furthermore, the low OR in both case groups when compared to the controls is evidence suggesting that low T4 indicates a lower chance of thyroid malignancy. The line created by the logistic regression is further evidence of a relationship between probability of cancer and serological level of fT4. In fact, using the regressed line gives a cut-off value which could prove to be a margin of safety in future studies.

Though the average amounts of fT4 are quite similar in both patients with malignant nodules and non-malignant nodules, 14.3 and 14.6 pmol/L respectively, the standard deviations are extremely different, 3.85 and 7.56 pmol/L. This relationship indicates that though the average patient with a cancerous thyroid nodule will have an fT4 value which seems normal, there are many more of them that deviate significantly from the mean as compared to benign nodules. Indeed this relationship remains true when comparing fT3 (average of 5.27 pmol/L with a standard deviation of 3.30 in cases of malignant nodules, average of 4.63 pmol/L with a standard deviation of 1.933 in cases of benign nodules).

No significant relationship between low fT3 and malignancy was identified in this study. The relationship has been shown in studies such as Jonklaas et al. [12]. Though this paper dealt only with total T3 and not fT3, it follows that if total T3 is low, fT3 should be as well. Interestingly, this is not in agreement with Cho et al. who have found no significant relationship between these variables, [11] or with Rinaldi et al. who non-significantly found high fT3 to be indicative of cancer risk [25]. Given that this study has not found any evidence in support of either group, it is possible that it was not sufficiently powered to find such a correlation, or that no relationship exists between the two.

Since fT4 tends to be high in cases of malignancy while fT3 tends to be low, a quotient (fT4/fT3) can be used to evaluate if these trends increase the risk of malignancy then either variable alone. In fact, there is a significant difference between cases and controls when comparing the average fT4/fT3 and the OR is quite high. This strong correlation indicates that even in cases where fT4 and fT3 appear normal, if the quotient is >3.3, a patient is 3.6 times more likely to harbour a malignant nodule. While the sensitivity is low, the specificity is high so such a calculation may help rule-in the possibility of thyroid cancer.

One theory which explains the relationship between high fT4 and low fT3 is that as malignancy develops in the thyroid, there is a disturbed expression of type 1 iodothyronine deiodinase [26]. This would lower the rate of conversion from the inactive fT4 state to the active fT3 state and explain the relationship found in this study. While the theory offers a potential explanation for this study’s findings, it remains only a theory and requires validation.

Sized categorization of thyroid nodule did not yield any statistically significant data, specifically for the T4/T3 quotient. This is likely because the study was not powered to detect differences in the quotient in size specific categories, as significance is only seen in the overall group.

There are several limitations to our study. First, its retrospective nature. Second, the cut offs of fT4 and fT3 fall into the higher or lower ends of the normal range. Additionally, groups were not matched for disorders or medications that may influence thyroid hormone levels. Prospective studies could focus on relating TSH, fT4 and fT3 values to indeterminate thyroid nodules.

## Conclusion

In this study, an elevated fT4/fT3 quotient (>3.3) increased the likelihood that a thyroid nodule was malignant. Malignant thyroid nodules may lose the ability to convert fT4 to fT3 as effectively as benign nodules, which may explain the findings uncovered in this study. Given that this is a retrospective analysis of a single patient cohort, further duplicate studies are required in distinct populations with larger sample sizes to evaluate the quotient’s validity. If the relationship is globally significant, future studies could relate fT4/fT3 to indeterminate thyroid nodules in order to see how the quotient could be helpful.

## Abbreviations

fT3: Free triiodothyronine
fT4: Free thyroxine
MTNS: McGill thyroid nodule score
OR: Odd’s ratio
T3: Triiodothyronine
T4: Thyroxine
TSH: Thyroid stimulation hormone
TT3: Total triodothyronine
US-FNA: Ultrasound guided fine-needle aspiration biopsy
